# Chromosome-level assembly and evolution analysis of the *Trichosanthes truncata* genome

**DOI:** 10.1038/s41597-024-03608-2

**Published:** 2024-08-12

**Authors:** Ying Hu, Xiaomei Wei, Zhuannan Chu, Fan Wei, Yude Peng, Baoyou Huang, Ling Dong, Kunhua Wei, Weiwen Li

**Affiliations:** 1 National Center for Traditional Chinese Medicine Inheritance and Innovation, Guangxi Botanical Garden of Medicinal Plants, Nanning, 530023 China; 2 Guangxi Key Laboratory of Medicinal Resources Protection and Genetic Improvement, Guangxi Botanical Garden of Medicinal Plants, Nanning, 530023 China; 3 Institute of Horticulture, Anhui Academy of Agricultural Sciences, Hefei, 230001 China

**Keywords:** Genome assembly algorithms, Plant evolution

## Abstract

*Trichosanthes truncata* C. B. Clarke, an important medicinal plant, is a dioecious plant belonging to the Cucurbitaceae family. This study presents a chromosomal-level reference genome assembly for *T. truncata*. Through the integration of PacBio high-fidelity sequencing and high-throughput chromosome conformation capture technology, a final genome sequence of 637.41 Mb was assembled, with an N50 of 57.24 Mb and consisting of 11 pseudochromosomes. Additionally, 97.21 Mb of repetitive sequences and 36,172 protein-coding genes were annotated. This high-quality genome assembly is of utmost significance for studying the molecular mechanisms underlying the biosynthesis of bioactive compounds. Furthermore, this study provided valuable insights into plant comparative genomics research.

## Background & Summary

*Trichosanthes*, which belongs to the family Cucurbitaceae, is one of the largest genera, most of which are annual or perennial herbs distributed in tropical Asia and Australia^1^. It has been reported that *Trichosanthes* ranks among the top five genera with considerable potential for discovery of anticancer agents out of the 145 families of plants^2^. *Trichosanthes* has been found to possess significant medicinal properties, including anti-inflammatory, antioxidant, hypoglycemic, and anticancer effects^1,2^.

**Fig. 1:**
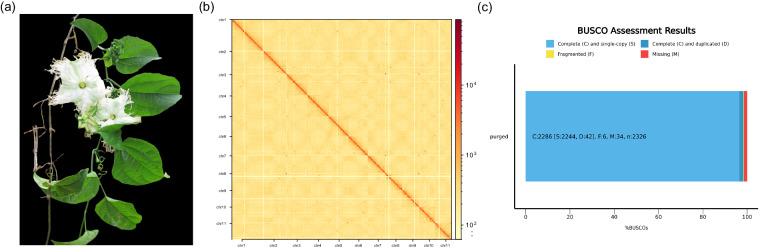
Genome assembly of *Trichosanthes truncata* C. B. Clarke. (**a**) Photo of *T. truncata* C. B. Clarke. (**b**) Hi-C heatmap showing chromosomal interactions under a resolution of 1 Mb. Only sequences anchored on chromosomes are shown. The abbreviations chr1-11 represent the 11 chromosomes, and the color bar represents the log2 value of interaction counts. (**c**) Completeness of the expected gene space of the genome assembly estimated with BUSCO.

*Trichosanthes truncata* C. B. Clarke, commonly known as the pointed leaf gourd, is a dioecious perennial vine herb belonging to the Cucurbitaceae family^3^ (Fig. 1a). *T. truncata* contains abundant organic Se and beneficial bioactive substances such as trichosantin and exhibits diverse effects such as coronary artery dilation, increased blood flow, reduced heart rate, enhanced hypoxia tolerance, acute myocardial ischemia resistance, and platelet aggregation inhibition^3,4^. Furthermore, its toxicity is lower than that of other *Trichosanthes* species, emphasizing its significant medicinal value^3,4^. In addition, most *Trichosanthes* species, including *T. truncata*, are dioecious^5^. In contrast, another *Trichosanthes* species, the snake gourd (*Trichosanthes anguina* L.), which belongs to the same genus and a fully assembled genome sequence is available, is monoecious and diclinous^6,7^.

Given the limited genomic resources of *T. truncata*, comprehensive insights into the genetic characteristics and functions associated with the production of bioactive compounds remain limited. We employed PacBio high-fidelity (HiFi) sequencing and high-throughput chromosome conformation capture (Hi-C) techniques to obtain chromosome-level reference genome sequences for *T. truncata*. In this study, we generated a high-quality genome assembly of *T. truncata*, which will greatly facilitate the elucidation of the molecular mechanisms underlying the biosynthesis of beneficial compounds with medicinal value in *T. truncata*, the exploration of comparative genomic research in the Cucurbitaceae family, thus providing a vital genomic resource and a foundation for further investigations.

## Methods

### Sample preparation and DNA sequencing

The voucher specimen of *T. truncata* was stored at Guangxi Botanical Garden of Medicinal Plants (http://www.gxyyzwy.com/, Ying Hu, hying@gxyyzwy.com), with a voucher number of YY10816. *T. truncata* fresh leaves were collected from the introduction and preservation nurseries in the Guangxi Botanical Garden of Medical Plants, and the samples were stored at 4 °C before being sent to Shenzhen BGI Genomic for sequencing. Total genomic DNA was extracted from the frozen leaves using cetyltrimethylammonium bromide (CTAB) buffer (incubation for 60 min at 65 °C) and was purified using phenol/chloroform/isopentyl (25:24:1), isopropyl alcohol, and ethanol precipitation. Purified DNA was resuspended in Tris–EDTA buffer for sequencing.

### Library construction and genome sequencing

The extracted DNA samples were subjected to Megaruptor shearing, resulting in fragment sizes within the target range of 15–20 kilobases (Kb). Subsequently, the DNA underwent damage repair, end repair, ligation with known adapters, enzymatic digestion, and size selection using the BluePippin system (Sage Science, Beverly, MA, USA), ultimately yielding dumbbell-shaped libraries. After quality control checks, these libraries were utilized for PacBio HiFi sequencing.

Two short-read sequencing libraries with insert sizes of 270 bp and 500 bp were constructed from the resulting high-quality DNA. DNA was subjected to fragmentation (Covaris, Woburn, MA, USA) and end repair, followed by adaptor ligation, which enabled the formation of circular DNA molecules and subsequent rolling-circle amplification to produce DNA nanoballs (DNBs). To prepare a Hi-C library, cells were treated with formaldehyde to cross-link DNA–protein or protein–protein complexes, which were then subjected to fragmentation, end repair, purification, and adaptor ligation. The short-read sequencing libraries and the Hi-C library were sequenced using the DNBSEQ platform (MGI, Shenzhen, China) in paired-end mode.

### Genome size estimation

The genome size of *T. truncata* was estimated based on k-mer counting of sequencing data using the k-mer analysis toolkit v2.4.2^8^, followed by genome assessment, computation of genome size, and heterozygosity estimation using genomescope2^9^.

### Genome assembly and assessment

We assembled PacBio HiFi reads using hifiasm v0.16.1 and validated the correctness of the k-mer peak positions^10^. Subsequently, we employed purge_dups v1.2.6 (https://github.com/dfguan/purge_dups) to further refine the p_ctg.gfa results by de-duplication and eliminating haplotigs with excessively low or high coverage generated during the assembly process. The Arima mapping pipeline (https://github.com/ArimaGenomics/mapping_pipeline) was used to align the Hi-C data with the assembled genome contig sequences. Scaffolding was performed using SALSA2^11^, followed by manual curation and refinement of the scaffolding using Juicebox (JBAT) (https://github.com/aidenlab/Juicebox).

The completeness and duplication levels of genome assembly were assessed throughout the process using BUSCO v5.2.2^12^. The Extensive de novo TE Annotator (EDTA) pipeline v2.0.1^13^ was employed to annotate TEs, which were then used to softmask the genome assembly with RepeatMasker v4.1.2^14^. Annotation of protein-coding genes was performed using a combination of homology-based searches and de novo predictions. First, protein sequences of 10 known species from the Cucurbitaceae family including *T. anguina*, *Citrullus lanatus*, *Luffa aegyptiaca*, *Cucurbita moschata*, *Siraitia grosvenorii*, *Momordica charantia*, *Gynostemma pentaphyllum*, *Cucumis sativus*, *Lagenaria siceraria*, and *Benincasa hispida*, were aligned to the *T. truncata* genome using diamond v2.0.15^15^. Subsequently, de novo gene prediction was performed using Funannotate pipeline v1.8.7 (https://github.com/nextgenusfs/funannotate) including AUGUSTUS^16^, GlimmerHMM v3.02^17^, and SNAP v2006-07-28^18^. Based on protein sequences from the 10 related species and the predicted gene models, the annotation data were combined to generate final consensus gene models using EVidenceModeler v1.1.1^19^, which were then assessed using BUSCO with the “embryophyta_odb10” ortholog set^12^. The tRNAs were predicted using tRNAscan-SE v2.0^20^, and ribosomal RNAs were identified using RNAmmer^21^. PANTHER families were predicted using the program InterProScan v.5.41–78.0^22^. Gene functional annotation was performed using the eggNOG-mapper software v2^23^ and the evolutionary genealogy of genes (eggNOG) database v5.0^24^.

### Comparative genome analyses

Gene family clustering analysis included 11 species from the Cucurbitaceae family, as well as rice and papaya: *T. truncata*, *T. anguina*, *Citrullus lanatus*, *Luffa aegyptiaca*, *Cucurbita moschata*, *Siraitia grosvenorii*, *Momordica charantia*, *Gynostemma pentaphyllum*, *Cucumis sativus*, *Lagenaria siceraria*, *Benincasa hispida*, *Oryza sativa*, and *Carica papaya*. For each gene, only the transcript with the longest coding region was retained and genes encoding proteins with fewer than 50 amino acids were excluded. After filtering the gene sets for each species, the remaining protein sequences were aligned using Diamond^15^, and low-quality alignments were filtered using a default similarity threshold of 95%. Subsequently, the alignment results were clustered using the proteinortho software to generate protein orthologs^25^.

To detect whole-genome duplication (WGD) events within the *T. truncata* genome, we employed the MCScanX to delineate syntenic blocks and subsequently computed the 4DTv for all gene pairs in each syntenic segment^26^.

Gene families with abnormally high or low gene count within a species were filtered out, and expansion and contraction analyses of gene families were performed using CAFE5^27,28^. Gene Ontology (GO) and Kyoto Encyclopedia of Genes and Genomes (KEGG) pathway enrichment analysis of the expanded and contracted gene families was performed with clusterProfiler v4.8.3^29^.

## Data Records

The assembly is available at Genbank under the accession number GCA_033996785.1^30^. The raw genome sequencing data (PacBio and DNBSEQ short reads) have been deposited to NCBI database under the SRP accession number SRP465770^31^ and SRR29025305^32^.

## Technical Validation

### Genome sequencing and assembly

PacBio HiFi sequencing and Hi-C techniques were used to generate a chromosome-scale assembly of *T. truncata* C. B. Clarke. The total length of the final assembly was 637.41 Mb, with a GC content of 33.82% (Table 1), which was close to the genome size estimated by 25-mer analysis (genome size of 557.50 Mb and heterozygosity of 1.217%). The numbers of contigs and scaffolds were 154 and 83, respectively, and contig N50 and scaffold N50 were approximately 56.40 Mb and 57.24 Mb, respectively (Table 1). Eleven chromosomes were generated by concatenating the contigs based on the Hi-C reads (Table 1), which is consistent with the findings of previous studies based on karyotype analysis^33^. The interaction signal intensity around the diagonal of the genome-wide Hi-C heatmap was stronger than that of the off-diagonal signals (Fig. 1b), indicating the high quality of the chromosome-level genome assembly.

**Table 1:** Statistics of the Assembly and Annotation of the *Trichosanthes Kirilowii* Maxim. Genome.

Estimated genome size	557.50 Mb
Heterozygosity	1.22%
Assembly	
Total length (Mb)	637.41
GC content (%)	33.82
Coting N50 (Mb)	56.40
Contig number	154
Scaffold N50 (Mb)	57.24
Scaffold number	83
Pseudochromosome number	11
BUSCO completeness score (%)	98.30
Annotation	
Total length of repeats (Mb)	97.21
Number of protein-coding genes	36172
Mean transcript length (bp)	1015.65
Mean coding sequence length (bp)	2999.48
Mean exon length	227.57

### Genome annotation

The total length of the repeats was 97.21 Mbp, accounting for 15.25% of the reference genome sequence (Table 1). A comprehensive approach was employed to perform genome annotation in *T. truncata*, combining homology-based searches and multiple prediction algorithms (AUGUSTUS, GlimmerHMM, SNAP). We identified 36,172 protein-coding genes across the genome assembly, with an average transcript length of 1,015.65 bp (Table 1). The average lengths of the coding sequence and exons were 2,999.48 bp and 227.57 bp, respectively (Table 1). BUSCO evaluation revealed a completeness score of approximately 98.30% based on the genes in the “Embryophyta _odb10” ortholog set (Fig. 1c and Table 1), indicating a high degree of completeness^30^. While these methods provided a robust initial annotation, integrating RNA-seq data in future studies will be pivotal for refining gene annotations and improving accuracy, thereby enhancing our understanding of gene function and genomic dynamics in *T. truncata*.

### Gene family analysis

**Fig. 2:**
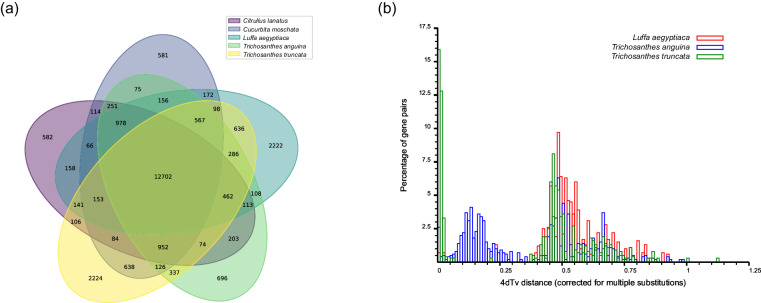
Gene family and evolution analysis. (**a**) Venn diagram representing a comparison of gene clusters among *T. truncata*, *C. lanatus*, *C. moschata*, *L. aegyptiaca*, and *T. anguina* L. (**b**) Detection of whole-genome duplication events of *T. truncata* by 4-fold degenerate synonymous sites (4DTv) comparisons.

Gene family cluster analysis of complete gene sets of *T. truncata* and 12 other sequenced plant genomes, including *T. anguina*, *C. lanatus*, *L. aegyptiaca*, *C. moschata*, *S. grosvenorii*, *M. charantia*, *G. pentaphyllum*, *C. sativus*, *L. siceraria*, *B. hispida*, *O. sativa*, and *C. papaya* was performed. All gene families were grouped into 37,061 gene clusters, with 1,060 single-copy orthologs. Further comparison of *T. truncata* with four closely related species (*C. lanatus*, *C. moschata*, *L. aegyptiaca*, and *T. anguina*) revealed 12,702 gene clusters shared by the five species. In addition, we found 2,224 gene families that were unique to the *T. truncata* genome when compared with the other four genomes (Fig. 2a).

### Comparative genome analysis

Fourfold degenerate synonymous site (4DTv) comparisons on gene synteny blocks revealed a recent *T. truncata*-specific whole-genome duplication (WGD) event (Fig. 2b). An expanded analysis of gene families was performed using 12 other sequenced plant genomes, which indicated that 158 gene families expanded in *T. truncata*, whereas 1140 gene families contracted, suggesting that more *T. truncata* gene families experienced contractions than expansions during adaptive evolution.

**Fig. 3:**
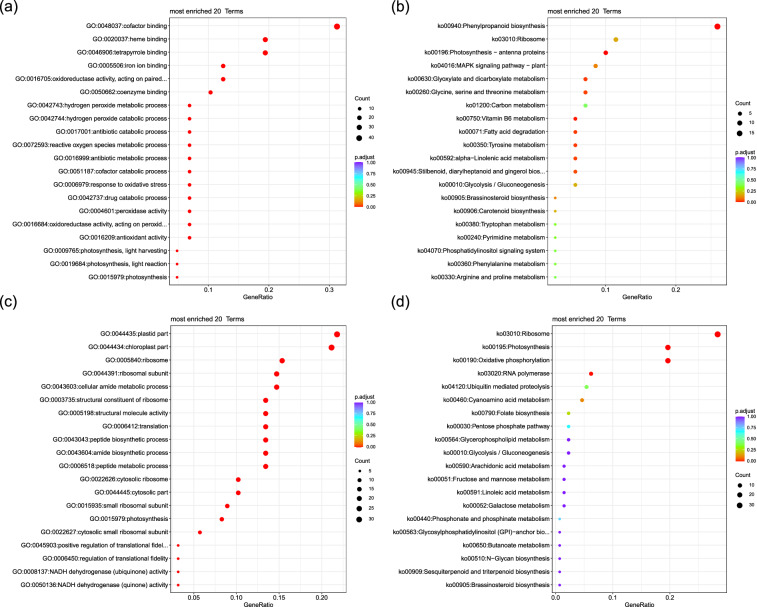
GO (**a,c**) and KEGG pathway (**b,d**) enrichment analyses of expanded (**a,b**) and contracted (**c,d**) gene families. The GO terms and KEGG pathways with an FDR-adjusted *p-value* < 0.05 were defined as statistically significant.

The expanded gene families were found to be primarily enriched in GO functions related to photosynthesis, including light reaction, light harvesting, antioxidant activity, oxidoreductase activity (acting on peroxide as an acceptor), oxidoreductase activity (acting on paired donors with the incorporation or reduction of molecular oxygen), peroxidase activity, hydrogen peroxide catabolic process, reactive oxygen species metabolic process, cofactor catabolic process, and antibiotic catabolic process (Fig. 3a). In terms of KEGG pathways, enrichment was observed in phenylpropanoid biosynthesis, Photosynthesis - antenna proteins, vitamin B6 metabolism, fatty acid degradation, tyrosine metabolism, glyoxylate and dicarboxylate metabolism, glycine, serine and threonine metabolism, alpha-linolenic acid metabolism, and stilbenoid, diarylheptanoid, and gingerol biosynthesis (Fig. 3b). On the other hand, the contracted gene families were mainly enriched in GO functions associated with chloroplast components, NADH dehydrogenase (quinone) activity, NADH dehydrogenase (ubiquinone) activity, positive regulation of translational fidelity, photosynthesis, amide biosynthetic process, and peptide biosynthetic process (Fig. 3c). In terms of KEGG pathways, enrichment was observed in photosynthesis, oxidative phosphorylation, ribosome, RNA polymerase, and cyanoamino acid metabolism (Fig. 3d).

## Data Availability

No specifc code was used in this study. The data analyses used standard bioinformatic tools, with parameters being clearly described in Methods. If specific parameters were not provided for the software, default settings recommended by the developer were utilized.
